# Synthesis and thermal decomposition kinetics of moisture curable polyurethane films as a reinforcing material for cultural relics

**DOI:** 10.1038/s41598-020-78705-4

**Published:** 2020-12-10

**Authors:** Xing Zhao, Liqin Wang, Lang Guo, Yanni Ma, Ziming Wang, Qing Niu, Liping Zheng

**Affiliations:** 1 Key Laboratory of Cultural Heritage Research and Conservation (Northwest University), Ministry of Education, Xi’an, People’s Republic of China; 2 School of Cultural Heritage, Northwest University, Xi’an, People’s Republic of China; 3 Xi’an Cultural Heritage Promotion Center, Xi’an, People’s Republic of China; 4 School of History and Society, Chongqing Normal University, Chongqing, People’s Republic of China

**Keywords:** Thermodynamics, Theory and computation

## Abstract

In situ consolidation is the most common treatment to conserve cultural relics, but materials for preserving fragile organic cultural relics in humid archaeological excavation sites are scarce. To solve the problem, a moisture-curable polyurethane (MCPU) prepolymer was synthesized by reacting isophorone diisocyanate with polyethylene glycol 600. The standard acetone–dibutylamine method, Fourier transform infrared spectroscopy, gel chromatography and thermogravimetric analysis were utilized to determine the change in isocyanate groups before and after the reaction, the prepolymer molecular weight, the thermal decomposition kinetic parameters and the MCPU film lifetime. The results showed that the number-average molecular weight of the prepolymer was 749, and the weight average molecular weight was 1684. Isophorone groups in the prepolymer react with moisture in the air to form colorless, transparent, flexible films. The thermal decomposition of the MCPU films was a first-order reaction, and the decomposition process consisted of two stages. The Dakin equation was used to obtain the thermal aging equation lg t = 4600.82/T − 8.07, meaning that at 15 °C, the sample has an approximately 150-year lifetime. A new conservation material was developed, and its thermal decomposition kinetics were studied, which are significant for the conservation of fragile organic cultural relics in humid environments.

## Introduction

The environment in archaeological excavation sites, such as tombs and grottoes, is typically very humid (relative humidity > 90%^1^). Organic cultural relics, having been buried in this environment for thousands of years, are rotten and cannot be extracted. To prevent further damage to these cultural relics, the most effective conservation method is the application of reinforcing materials.

A wide variety of reinforcing materials for cultural relics are available, and the most commonly used organic materials, include Paraloid B72 (B72)^2–5^, polyvinyl butyral (PVB)^6^, epoxy resin^7^, organosilicone^8,9^, etc. These materials are generally employed in a normal-humidity environment amenable to physical or chemical reactions that provide long-term protection for cultural relics. In a high-humidity environment, B72 and PVB whiten easily^10,11^, which can change the appearance of cultural relics, whereas epoxy resins^12^ and organosilicones are generally used for masonry preservation and are rarely implemented in the reinforcement of fragile organic cultural relics. Cyclododecane and menthol are new types of temporary consolidants for cultural relics^13^ that have melting points that are slightly above room temperature and can sublimate at room temperature. Solidification proceeds via the following steps. The consolidant is first melted under heat (or dissolved by a solvent) and applied to the surface of a cultural relic. When the temperature reduces to room temperature (solvent volatilization), the consolidant undergoes a phase change into a solid by adhering to the cultural relic. By the time the fragile cultural relic is safely extracted and transferred to the laboratory, the consolidant typically leaves the substrate unaided. Thus, the fragile organic cultural relics need to be reinforced again, which may result in secondary damage. Currently, there are no suitable materials for the reinforcement and conservation of fragile organic cultural relics in high-humidity environments. Furthermore, there are few reports on the thermal decomposition kinetics of reinforcing materials for cultural relics^14^.

Moisture-curable polyurethane (MCPU) is a polyurethane that contains isocyanate end groups and is widely used in waterproof coatings for buildings, anti-corrosion materials, bio-pharmaceutical materials, electronic materials, wood adhesives, etc.^15^. However, this material has rarely been used for the conservation of cultural relics. The principle of MCPU reinforcement is based on the chemical reaction between terminal isocyanate groups and the water in the atmosphere, such as is available in the high-humidity environments in which cultural relics are found. In this paper, a MCPU prepolymer was synthesized for use in high-humidity environments, and the thermal decomposition kinetics of the MCPU films were studied, providing a scientific basis for the selection of cultural relic reinforcement materials and research on the aging process of these materials.

## Results and discussion

### Synthesis of MCPU prepolymer

PEG600 possesses excellent flexibility and hydrolysis resistance, and IPDI has excellent resistance to yellowing. Using DBTDL as a catalyst, the isocyanate-terminated polyurethane prepolymer was synthesized by a nucleophilic addition reaction.

The isocyanate index (R value) is the molar ratio of isocyanate groups to alcoholic hydroxyl groups in the reactants. This ratio affects the molecular weight and structure of the prepolymer as well as the properties of the cured films, thus affecting the reinforcement and conservation of cultural relics. For R values > 1, the end groups of the prepolymer molecules are isocyanates. The higher is the R value, the more available are the isocyanate groups in the system, the shorter is the curing time, and the more rigid is the cured film: the converse also holds. After pre-experimental screening, the R value was determined to be 3 based on several indicators, such as the prepolymer performance, the curing time, and the properties of the cured film.

**Figure 1:**
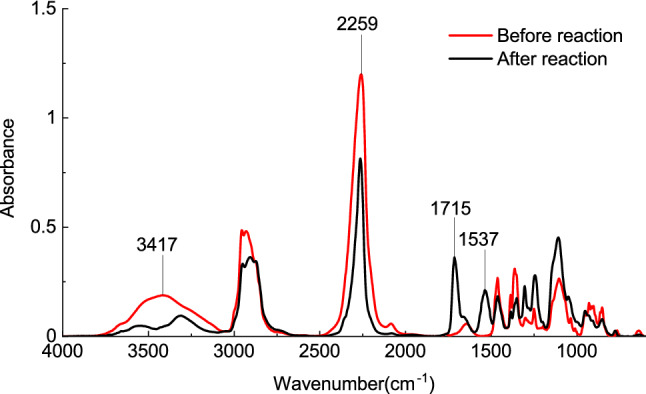
FTIR spectra of the reaction mixture before and after the reaction.

A titration experiment showed that the content of isocyanate groups was 19.81% before the reaction and 13.18% at the end of the reaction. Therefore, one-third of the isocyanate groups reacted with hydroxyl groups to form urethane groups, and the remaining two-thirds were unreacted. The FTIR absorption spectra (Fig. 1) showed that the contents of hydroxyl groups at 3417 cm^−1^ and isocyanate groups at 2259 cm^−1^ were reduced in the product and were accompanied by new absorption peaks at 1715 cm^−1^ and 1537 cm^−1^, indicating the formation of urethane groups. The GLC results showed that the weight average molecular weight was 1684 and the number average molecular weight of the prepolymer was 749, which is approximately 1% of the molecular weight of the most commonly used consolidant B72^16^. The use of MCPU for the reinforcement of cultural relics offers the following advantages: the low molecular weight helps enhance the permeability of the reinforcing material, and the relatively high polydispersity index (PDI) ensures that prepolymer molecules with different molecular weights can continuously penetrate to different depths, thereby preventing damage from variations in the stress in between layers^17^ to achieve long-term conservation.

When the MCPU prepolymer is used as a reinforcing agent in cultural relic protection, the isocyanate groups react with the water molecules in the atmosphere to form amine groups and release carbon dioxide. These amine groups interact with unreacted isocyanate groups to form ureido groups and eventually solidify into polyurethane and polyurea structures. The cured film is colorless, transparent, and flexible and does not affect the appearance of cultural relics after reinforcement.

### Thermal decomposition kinetics of MCPU films

**Figure 2:**
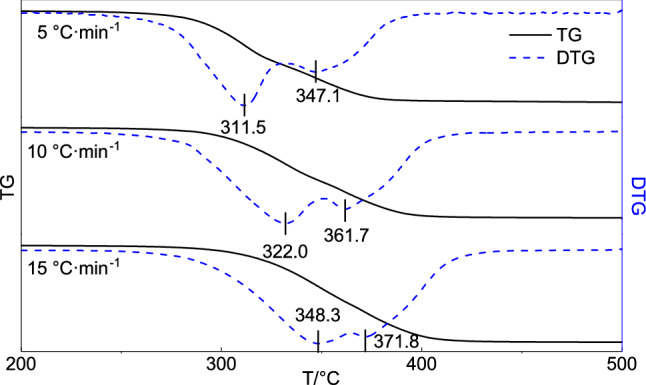
TG-DTG curves of MCPU films.

The reinforcement of fragile organic cultural relics is usually irreversible, thus reinforcement materials must exhibit adequate thermal stability. Thermal analysis is an effective means of measuring the thermal stability of solid materials in terms of the thermal decomposition temperature, thermal decomposition kinetics, and the material lifetime^18,19^. The TG and differential TG (TG-DTG) curves of the synthesized MCPU films at heating rates of 5, 10, and 15 °C·min^−1^ are shown in Fig. 2. The corresponding temperatures for the different conversion rates (α) are shown in Table 1. In Fig. 2, two weight-loss stages can be observed for the different heating rates ranging from 311.5–348.3 to 347.1–371.8 °C. As the temperature increases in the thermal analysis process, molecular chain motion gradually intensifies into chain scission, which is manifested as a sustained weight loss. When the heating rate increases, the relaxation of molecular chain motion cannot keep pace with the time scale of the experimental measurement, which manifests as a shift in the weight-loss peak towards high temperatures.

**Table 1:** Relationship between conversion rate and temperature for three heating rates.

α	T(°C)		
	5 °C·min^−1^	10 °C·min^−1^	15 °C·min^−1^
0.05	259.60	278.34	289.02
0.10	281.52	296.73	307.94
0.20	295.75	311.76	325.43
0.30	304.31	321.85	336.45
0.40	311.22	330.24	345.30
0.50	318.16	338.34	353.64
0.60	328.16	348.13	362.49
0.70	340.00	358.74	371.76
0.80	350.55	368.40	381.15
0.90	361.90	379.87	392.85

#### Reaction order

Most decomposition reactions can be approximated as first-order reactions (n = 1)^20^. According to the Coats-Redfern method^21^, when n = 1,

1 $${\text{lg }}\left[ { - {\text{lg }}\left( {1 - \alpha } \right)/T^{2} } \right] = \left( {1 - 2RT/E} \right){\text{ lg }}\left( {{\text{AR}}/{\beta E}} \right) - E/2.303RT$$

where α is the conversion rate, T denotes the temperature (K), and β denotes the heating rate (°C·min^−1^), A is the pre-exponential factor (min^−1^), E denotes the activation energy (J·mol^−1^), and R is the ideal gas constant.

If plotting lg [− lg (1-α)·T^−2^] versus 1/T yields a straight line, the reaction is considered to be first order, and the slope equals − E/2.303R. However, if the lower part of the plot deviates from a straight line, then the reaction is not first order.

**Figure 3:**
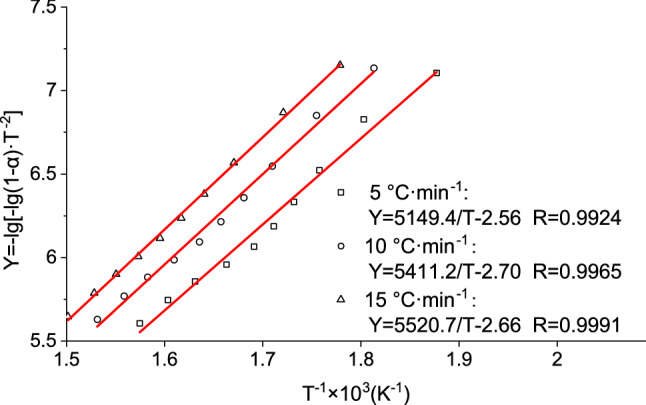
Typical Coats-Redfern kinetic plots for the thermal decomposition of MCPU films.

In Fig. 3, the plots of Y = − lg [− lg (1 − α)·T^−2^] vs. 1/T at the different heating rates are straight lines with correlation coefficients R > 0.99. This result indicates that the thermal decomposition of MCPU was independent of the heating rate. For heating rates of 5, 10, and 15 °C·min^−1^, the thermal decomposition matched a first-order reaction.

#### Activation energy at different conversion rates

With linear heating, the reaction kinetic equation can be expressed as:

2 $$\frac{{{\text{d}}\alpha }}{{{\text{dt}}}} = f\left( \alpha \right)k\left( T \right)$$

3 $$\frac{{{\text{d}}\alpha }}{{{\text{dT}}}} = \frac{1}{\beta }f\left( \alpha \right)k\left( T \right)$$

where t denotes the reaction time, f(α) is the kinetic model for the reaction, and k(T) is a temperature-dependent rate constant that can be expressed by the Arrhenius equation:

4 $${\text{k}}\left( {\text{T}} \right) = {\text{Aexp}}\left( { - {\text{E}}/{\text{RT}}} \right)$$

Substituting Eq. (4) into Eq. (3) and integrating the variables separately yields:

5 $$\mathop \smallint \limits_{{\upalpha }_{0} }^{\alpha } \frac{d{\upalpha }}{f\left( {\upalpha } \right)} = \frac{A}{\upbeta }\mathop \smallint \limits_{T_{0} }^{T} {\text{exp}}\left( { - {\text{E}}/{\text{RT}}} \right)dT$$

where α_0_ is the conversion rate at T = T_0_.

At the initial reaction time T_0_, when the temperature is relatively low, and the reaction rate is slow, Eq. (5) can be approximated as:

6 $$\mathop \smallint \limits_{0}^{\alpha } \frac{d{\upalpha }}{f\left( {\upalpha } \right)} = \frac{A}{\upbeta }\mathop \smallint \limits_{0}^{T} {\text{exp}}\left( { - {\text{E}}/{\text{RT}}} \right)dT$$

The kinetic model for a first-order reaction^22^ can be expressed as:

7 $${\text{f}}\left( {\upalpha } \right) = 1 - {\upalpha }$$

Doyle’s approximation can be used to express Eq. (6) as:

8 $${\text{lg }}\beta = {\text{lg }}\left[ { - AE/Rln\left( {1 - \alpha } \right)} \right] - 2.315 - 0.4567E/RT$$

Equation (8) shows that at a fixed conversion rate, the plot of lg β vs. 1/T is a straight line. The decomposition activation energy can be derived from the slope of the straight line, and the pre-exponential factor A can be obtained from the intercept (Table 2). Table 2 shows that the thermal decomposition activation energy was in the 88.09–108.57 kJ·mol^−1^ range.

**Table 2:** Regression equations of lg β against 1/T, activation energies and pre-exponential factor.

α	lg β vs. 1/T			E (kJ mol^−1^)	A (min^−1^)
	Slope	Intercept	R		
0.05	− 4838.82	9.78	1.00	88.09	6.02 × 10^6^
0.10	− 5851.66	11.25	1.00	106.53	3.05 × 10^8^
0.20	− 5503.41	10.38	1.00	100.19	9.26 × 10^7^
0.30	− 5255.81	9.81	1.00	95.68	4.14 × 10^7^
0.40	− 5503.41	10.38	1.00	100.19	2.12 × 10^8^
0.50	− 5255.81	9.81	1.00	95.68	8.04 × 10^7^
0.60	− 5089.17	9.42	1.00	92.65	4.42 × 10^7^
0.70	− 5010.85	9.18	1.00	91.22	3.43 × 10^7^
0.80	− 5335.51	9.58	1.00	97.14	1.07 × 10^8^
0.90	− 5963.44	10.43	1.00	108.57	9.77 × 10^8^

#### Lifetime calculation

After deployment, aging reactions continue to occur in the MCPU films. Dakin proposed the following empirical formula for material aging:

9 $$\lg t = a/{\text{T}} + b$$

where T denotes the environmental temperature (K), t denotes the lifetime (min) at temperature T, and a and b are constants.

From Eqs. (2), (4), and (7), we have:

10 $$\mathop \smallint \limits_{0}^{\alpha } \frac{1}{1 - \alpha }d\alpha = \mathop \smallint \limits_{0}^{t} {\text{Aexp}}\left( { - {\text{E}}/{\text{RT}}} \right)dt$$

Integrating Eq. (10) yields:

11 $$\lg t = E/2.303RT + {\text{lg }}\left[ { - \ln \left( {1 - \alpha } \right)/A} \right]$$

Substituting Eq. (9) into Eq. (11) yields:

12 $$a = E/2.303R$$

13 $$b = {\text{lg }}\left[ { - \ln \left( {1 - \alpha } \right)/A} \right]$$

In material thermal stability research, mass loss is often used to characterize material aging. Setting a mass loss (conversion) of 5% as the lifetime and substituting E = 88092.6 J·mol^−1^, A = 6.02 × 10^6^ min^−1^ and Eqs. (12) and (13) into Eq. (9), yields the aging formula:

14 $$\lg t = 4600.82/{\text{T}} - 8.07$$

**Figure 4:**
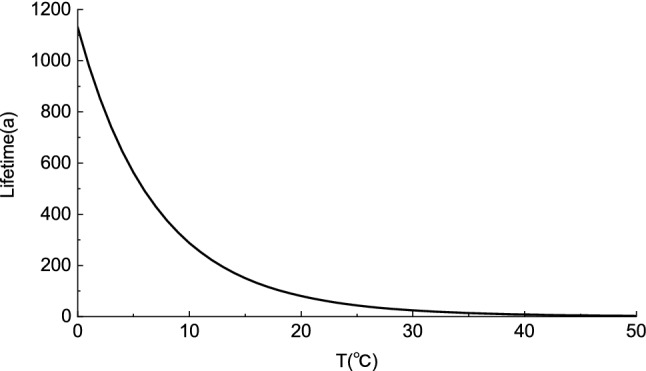
Relationship between MCPU lifetime and ambient temperature.

The relationship between the temperature and lifetime is shown in Fig. 4. At low temperatures, the lifetime of the material decreased rapidly with increasing temperature. The ambient temperature of a museum warehouse is generally low. For an ambient temperature of 15 °C, the lifetime of the MCPU film in nitrogen was determined to be over 150 years, which is higher than that of the Class A standard for conservation materials of cultural relics (100 years)^23^. A previous study^24^ has shown that a conservation material can undergo oxidative crosslinking in air, thereby increasing the activation energy of the decomposition process and enhancing the stability of the material. Therefore, the MCPU film meets the stability requirements for conservation materials of cultural relics.

## Conclusion

An isocyanate-terminated polyurethane prepolymer is synthesized and used as a reinforcing material for fragile organic cultural relics located in a high-humidity environment. The number average molecular weight of the prepolymer is 749. After solidification, a colorless, transparent, and flexible thin film is obtained. At heating rates of 5, 10, and 15 °C·min^−1^, the thermal decomposition of the MCPU thin film exhibits first-order reaction kinetics. The MCPU thin film exhibits two weight-loss stages from 311.5–348.3 °C and 347.1–371.8 °C. The activation energy ranges from 88.09 to 108.57 kJ·mol^−1^. The thermal lifetime equation is obtained as lg t = 4600.82/T-8.07. Consequently, the material lifetime is longer than 150 years at 15 °C. This study addresses the scarcity of reinforcing materials for fragile organic cultural relics situated in high-humidity environments. A comparative aging study on MCPU films in air and other natural environments is suggested as future research.

## Methods

### Instruments and reagents

Analyses were performed using a LUMOS Fourier transform infrared (FTIR) spectroscopy (Bruker, Germany), UltiMate 3000 gel chromatography (GLC, Dionex, USA) and a thermogravimetry/differential scanning calorimetry (TG/DSC) 3 + synchronous thermal analyzer (Mettler Toledo, Switzerland).

Polyethylene glycol 600 (PEG600), isophorone diisocyanate (IPDI, 99%) and dibutyltin dilaurate (DBTDL) were purchased from the Aladdin reagent company. Ethyl acetate (AR), acetone (AR), and di-n-butylamine (AR) were purchased from the Sinopharm Chemical Reagent Company.

### Synthesis of MCPU prepolymer

On the basis of our preliminary research^17,25^, the synthesis conditions were further optimized as follows. Dehydrated PEG600 and IPDI (in a 1:3 molar ratio) were added to a three-necked flask and preheated at 60 °C for 10 min. Then, 0.5% DBTDL (as a proportion of the entire reaction system mass) was added to the flask. After stirring under nitrogen for 3 h, the MCPU prepolymer was obtained as a white viscous product. The product was diluted with ethyl acetate solvent, and the resulting solution was sealed and stored.

A total of 3 mL of MCPU prepolymer ethyl acetate solution (corresponding to a mass fraction of 20%) was evenly coated on 25 mm × 75 mm glass slides and solidified for 24 h at 100% relative humidity. The films were peeled off for later use.

### Characterization of MCPU films

The standard acetone-dibutylamine titration method^26^ was used to determine the content of isocyanate groups in the synthetic system. The FTIR absorption spectra of the samples were determined by the KBr tableting method with the FTIR microscope; the wavenumber range was 4000–600 cm^−1^, and the resolution was 4 cm^−1^. The molecular weights of the prepolymers were measured by GLC with tetrahydrofuran as the mobile phase. A synchronous thermal analyzer was used to perform a thermal analysis of the sample under a nitrogen atmosphere at a heating rate of 5–15 °C·min^−1^ over a temperature range of 50–500 °C.

## Data Availability

Part of the data generated or analyzed during this study are included in this published article and its supplementary information files. The rest of the datasets used and/or analyzed during the current study are available from the corresponding author on reasonable request.

## References

[1] Ruffolo SA (2017). Medium-term in situ experiment by using organic biocides and titanium dioxide for the mitigation of microbial colonization on stone surfaces. Int. Biodeterior. Biodegrad..

[2] Fantoni R (2013). Laser-induced fluorescence study of medieval frescoes by Giusto de’ Menabuoi. J. Cult. Herit..

[3] Cataldi A, Deflorian F, Pegoretti A (2015). Microcrystalline cellulose filled composites for wooden artwork consolidation: application and physic-mechanical characterization. Mater. Desig..

[4] Liccioli L, Fedi M, Carraresi L, Mandò PA (2017). Characterization of the chloroform-based pretreatment method for 14C dating of restored wooden samples. Radiocarbon.

[5] Muhcu D, Terzi E, Kartal SN, Yoshimura T (2017). Biological performance, water absorption, and swelling of wood treated with nano-particles combined with the application of Paraloid B72^®^. J. Forest. Res..

[6] France CA, Giaccai JA, Doney CR (2015). The effects of Paraloid B-72 and Butvar B-98 treatment and organic solvent removal on delta(13)C, delta(15)N, and delta(18)O values of collagen and hydroxyapatite in a modern bone. Am. J. Phys. Anthropol..

[7] Sideridou ID, Vouvoudi EC, Papadopoulos GD (2016). Epoxy polymer Hxtal NYL-1^TM^ used in restoration and conservation: irradiation with short and long wavelengths and study of photo-oxidation by FT–IR spectroscopy. J. Cult. Herit..

[8] Xu F (2015). Preparation of modified epoxy–SiO_2_ hybrid materials and their application in the stone protection. Prog. Org. Coating..

[9] Formia A, Tulliani J-M, Antonaci P, Sangermano M (2014). Epoxy monomers consolidant for lime plaster cured via a redox activated cationic polymerization. J. Cult. Herit..

[10] Lettieri M, Masieri M (2016). Performances and coating morphology of a siloxane-based hydrophobic product applied in different concentrations on a highly porous stone. Coatings.

[11] Wang, B. *Applicability study of the reinforcement material of the commonly used relics in different humidity environments*, M. S. dissertation, Northwest University, Xi'an, China (2017).

[12] Tesser E, Lazzarini L, Bracci S (2018). Investigation on the chemical structure and ageing transformations of the cycloaliphatic epoxy resin EP2101 used as stone consolidant. J. Cult. Herit..

[13] Han X (2014). The use of menthol as temporary consolidant in the excavation of Qin Shihuang's Terracotta army. Archaeometry.

[14] Du W, Yang C, Zhang B, Rong B, Zhou T (2018). Exploratory research on prediction of effective lifetimes of several typical materials used for the conservation of polychrome potteries. Sci. Conserv. Archaeol..

[15] Casdorff K (2018). About the influence of a water-based priming system on the interactions between wood and one-component polyurethane adhesive studied by atomic force microscopy and confocal Raman spectroscopy imaging. Int. J. Adhes. Adhes..

[16] Conti C, Striova J, Aliatis I (2013). Portable Raman versus portable mid-FTIR reflectance instruments to monitor synthetic treatments used for the conservation of monument surfaces. Anal. Bioanal. Chem..

[17] Zhao X (2017). Synthesis and application of moisture curable polyurethane as the consolidant for cultural relics. Chem. Reagent..

[18] He T, Yue K-F, Chen S-P, Zhou C-S, Yan N (2016). Synthesis, structure and thermodynamics/kinetics analysis of three different interpenetrating zinc (II) coordination architectures. Acta Phys. Chim. Sin..

[19] Yu H-Y, Wang F, Liu Q-C, Ma Q-Y, Gu Z-G (2017). Structure and kinetics of thermal decomposition mechanism of novel silk fibroin films. Acta Phys. Chim. Sin..

[20] Run M, Zhang D, Wu S, Wu G (2007). Thermal decomposition of poly(ethylene terephthalate)/mesoporous molecular sieve composites. Front. Chem. Eng. China.

[21] Coats AW, Redfern JP (1964). Kinetic parameters from thermogravimetric data. Nature.

[22] Chandrasekaran A, Ramachandran S, Subbiah S (2017). Determination of kinetic parameters in the pyrolysis operation and thermal behavior of *Prosopis juliflora* using thermogravimetric analysis. Biores. Technol..

[23] Feller, R. L. *Accelerated aging: photochemical and thermal aspects* (Getty Publications, 1995).

[24] Wang H, Yang J, Zhou P, Long S, Du Z (2004). Studies on the thermal lifetime of poly (phenyene sulfide sulfone. Polym. Mater. Sci. Eng..

[25] Zhao X (2018). Synthesis, testing and application of moisture-curable polyurethane as a consolidant for fragile organic cultural objects. J. Adhesion Sci. Tech..

[26] Hu Y, Liu C, Shang Q, Zhou Y (2017). Synthesis and characterization of novel renewable castor oil-based UV-curable polyfunctional polyurethane acrylate. J. Coating. Tech. Res..

